# Tracking slab surface temperatures with electrical conductivity of glaucophane

**DOI:** 10.1038/s41598-021-97317-0

**Published:** 2021-09-09

**Authors:** Geeth Manthilake, Ye Peng, Kenneth T. Koga, Mainak Mookherjee

**Affiliations:** 1 Laboratoire Magmas et Volcans CNRS, IRD, OPGC, Université Clermont Auvergne, 63000 Clermont-Ferrand, France; 2 Earth Materials Laboratory, Department of Earth, Ocean and Atmospheric Sciences, Florida State University, Tallahassee, FL 32306 USA

**Keywords:** Petrology, Mineralogy

## Abstract

Slab surface temperature is one of the key parameters that incur first-order changes in subduction dynamics. However, the current thermal models are based on empirical thermal parameters and do not accurately capture the complex pressure–temperature paths of the subducting slab, prompting significant uncertainties on slab temperature estimations. In this study, we investigate whether the dehydration-melting of glaucophane can be used to benchmark the temperature in the slab. We observe that dehydration and melting of glaucophane occur at relatively low temperatures compared to the principal hydrous phases in the slab and produce highly conductive Na-rich melt. The electrical properties of glaucophane and its dehydration products are notably different from the hydrous minerals and silicate melts. Hence, we conclude that the thermodynamic instability of glaucophane in the slab provides a unique petrological criterion for tracking temperature in the present-day subduction systems through magnetotelluric profiles.

## Introduction

Upon subduction, hydrous mineral phases in a subducting slab reach their limits of thermodynamic stability and produce a continuous flux of mobile phases, i.e. aqueous fluids, silicate melts, or supercritical fluids, into the overlying mantle through multi-phase, multi-variant dehydration reactions^1^. The migration of mobile phases into the overlying mantle metasomatizes the mantle wedge^2,3^, triggers partial melting in the peridotite mantle^4^, and plays a crucial role in recycling volatiles^5–8^, and trace elements in subduction zones^9^.

Among numerous prograde metamorphic dehydration reactions, the transition from blueschist facies to eclogite facies has been considered the most significant, as it characterizes the transformation of altered and less dense oceanic crust into an anhydrous and dense slab^10^. A consensus view of the geoscience community is that the blueschist-eclogite transition has a depth range that locates beneath arc volcanoes^11^ and is considered as the principal driving force behind the fluid-assisted melting in the mantle wedge^10,12,13^.

The variations in slab temperature, composition, and subduction dynamics make each subduction zone unique and the differences are often reflected in the seismic velocity profiles, arc magmatism, and seismicity^10,14^. Slab temperature is one of the governing parameters that define the stability of hydrous minerals, and therefore magmatism of arc volcanoes. The subduction zone temperature profiles are estimated primarily based on numerical models, which use finite element analyses with a range of input parameters^10,15,16^. Geochemical constraints such as the H_2_O/Ce ratio in slab fluid compositions have also been used to predict slab surface temperature^17,18^. The H_2_O/Ce thermometer primarily depends on the chemical analyses of melt inclusion found in arc magmas. However, it has been observed that the degassing alters the H_2_O/Ce ratio by losing the primitive H_2_O even in inclusions^19^. While the existing models provide insights into the complex interplay of geophysical and geochemical parameters of the thermal structures of subduction zones, the lack of temperature-fixed points makes it harder to evaluate the accuracy of subduction zone temperature profiles. As a result, some thermal models report a geothermal gradient (dT/dz) < 5 K/km, which is unlikely for subduction zones^20^. This suggests that although the present parametrizations in thermal models are internally consistent, they may not adequately estimate the realistic subduction zone conditions^21^. A temperature-fixed point in the subducting slab, if it can be constrained with an independent observation, is crucial for constructing geodynamic models and improving the understanding of mantle flow within subduction zones.

Glaucophane (Na_2_(Mg_3_Al_2_)Si_8_O_22_(OH)_2_) and lawsonite (CaAl_2_Si_2_O_7_(OH)_2_.H_2_O) are the principal hydrous mineral phases associated with the blueschist facies lithology^1,22,23^. The presence of glaucophane and lawsonite is often associated with anomalous geophysical observations including a relatively low-velocity layer^24,25^ and enhanced electrical conductivity^26,27^ at the top of subducting slabs. The stability of lawsonite at high pressures (> 9 GPa) and high temperature up to 1300 K means that the lawsonite potentially remains stable to depths of more than 200 km^22,23^. In contrast, glaucophane is thermodynamically stable at lower P–T conditions and its dehydration marks the onset of the transition of hydrous oceanic crust to the dense anhydrous eclogite^10^. Notably, it has been observed that amphibole stability can be enhanced beyond 5 GPa by the substitution of fluorine for amphibole hydroxyl sites. Hence, depending on F contents, the blueschist-eclogite transition may shift to depths beyond 75 km^28,29^.

It is well established that the electrical conductivity of fluids or melts can be enhanced by the highly mobile Na^+^ as a charge carrier^30^. Because glaucophane is one of the principal hosts of Na in subducting slabs, we focus on understanding whether the dehydration-melting of glaucophane can generate distinct geophysically detectable electrical anomalies, standing out from the hydrous minerals, fluid, and melts in subduction zones. We investigate the electrical conductivity of glaucophane in situ under high pressure and high temperature up to 6 GPa and 1258 K. We show that the dehydration melting of glaucophane produces highly conductive Na-rich fluids and melt that can serve as geophysical indicators for accurately benchmarking the slab temperatures.

## Results

**Figure 1:**
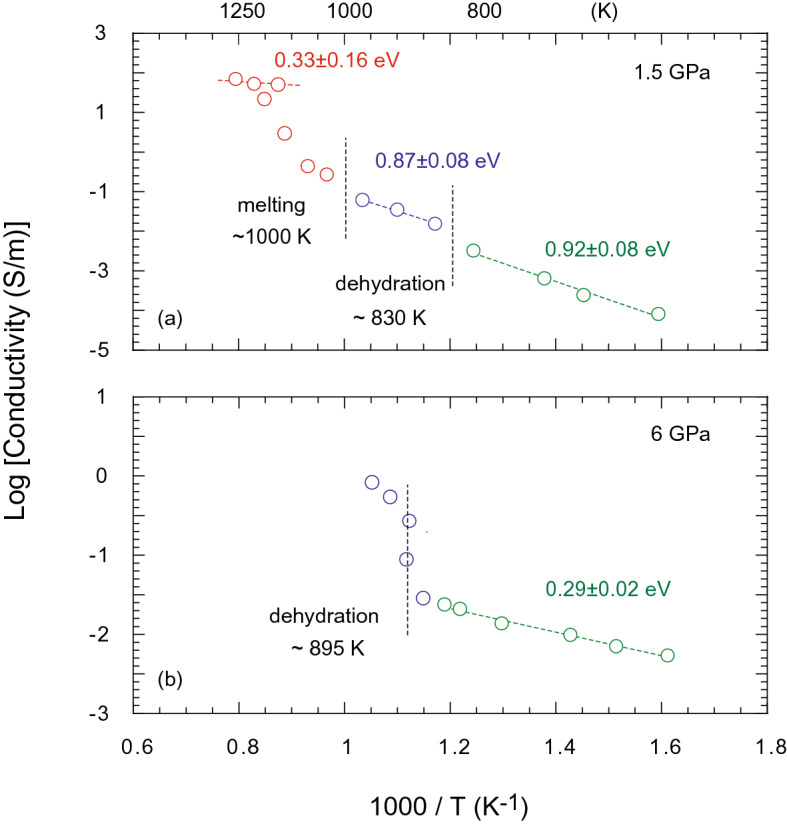
The electrical conductivity of glaucophane as a function of reciprocal temperature. **(a)** At 1.5 GPa, electrical conductivity is characterized by three stages, separated by discontinuous increases of conductivity at ~ 825 K and 1000 K. **(b)** Electrical conductivity of glaucophane at 6 GPa. The dehydration of the sample occurs at 895 K. The activation enthalpies corresponding to the dominant conduction mechanism at each stage are shown next to the fitting lines. The experimental uncertainty can arise from the estimation of temperature, geometrical parameters. Fitting errors are estimated to be less than 5% and are smaller than the symbol size.

**Figure 2:**
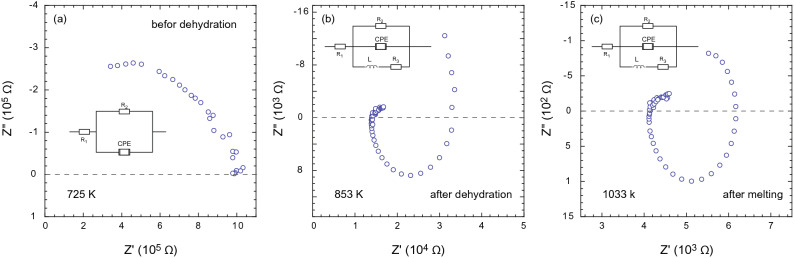
Impedance spectra of the glaucophane at 1.5 GPa. **(a)** At 725 K, below dehydration temperature of glaucophane (830 K), the impedance arc resembles grain interior processes, which can be modeled with a resistor- constant phase element (R-CPE) circuit. **(b)** At 853 K, the impedance spectrum shows the development of an inductive loop in response to the dehydration and the presence of fluid in the sample. The equivalent circuit can be represented as an R-CPE with an inductive component (L). The induction at low frequencies can be explained by the adsorption of ionic species in the electrode surface or erosion of electrodes due to fluid phases. **(c)** Further increase in temperature to 1033 K results in a sudden decrease in sample resistance. The persisting induction loop in impedance spectra indicates a possible reaction of melt with Ni electrodes.

The electrical conductivity of glaucophane increases discontinuously with increasing temperature (Fig. 1). At 1.5 GPa, two electrical conductivity discontinuities define three distinct conduction mechanisms in glaucophane corresponding to (i) the solid-state sample prior to dehydration (0.92 ± 0.08 eV), (ii) sample and aqueous fluid following dehydration (0.87 ± 0.08 eV), and (iii) partially molten sample (0.33 ± 0.16 eV) (Fig. 1a). The first discontinuous increase of conductivity to 0.1 S/m occurs at 830 K, which may correspond to glaucophane dehydration and the release of aqueous fluids. The second increase is observed at around 1000 K, possibly due to the melting of glaucophane and the production of a Na-rich melt. The sample exhibits electrical conductivity of 97 S/m at 1258 K. The electrical conductivity of glaucophane at 6 GPa exhibits a sharp increase of conductivity at around 895 K, which corresponds to the breakdown of glaucophane and the release of aqueous fluids in the sample. At 950 K, the sample exhibits electrical conductivity of 1 S/m (Fig. 1b). The impedance spectra of the samples display spectral features that are characteristic of dehydration and melting of a hydrous mineral^7,26^ (Fig. 2).

**Figure 3:**
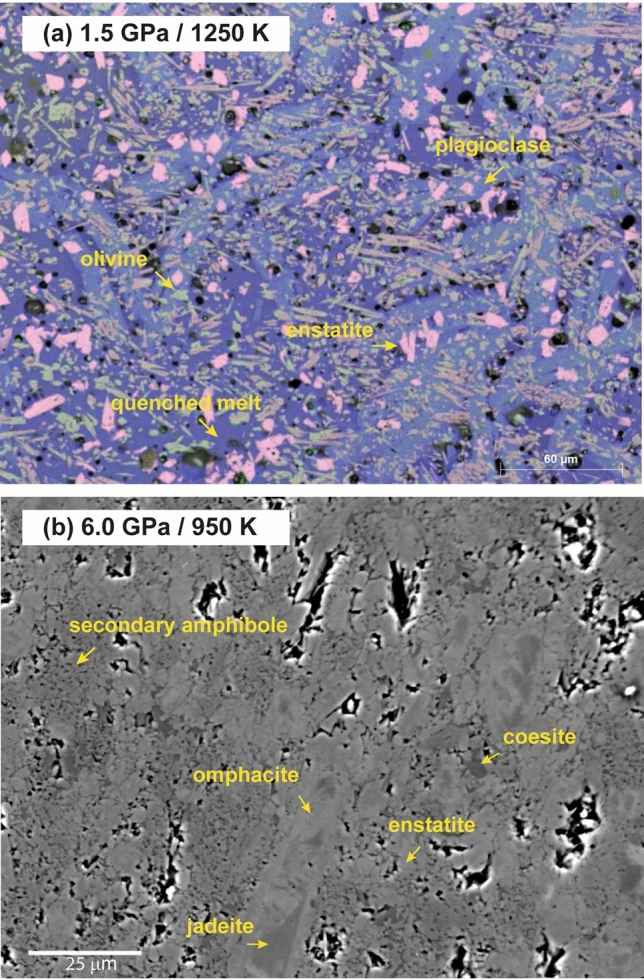
Back-scattered electrons (BSE) image of the recovered sample after the electrical conductivity measurements. (**a**) Energy-dispersive X-ray spectroscopy (EDS) compositional map overlapped with a back-scattered electrons (BSE) image of the recovered sample at 1.5 GPa. The phases present in the sample are olivine (green), enstatite (pink), plagioclase (light blue), and melt (indigo). (**b**) The BSE image showing the glaucophane starting sample recovered after electrical conductivity measurements at 6 GPa.

The observed conductivity variations correspond to textural and chemical changes of the sample, which confirms the dehydration and melting of glaucophane (Fig. 3). The chemical analyses of the sample after the electrical conductivity measurements are reported in Supplementary Table S1. Previous studies find that the dehydration of glaucophane at high temperature and high pressure follows the reactions: (1) glaucophane = jadeite + enstatite + nyböite + H_2_O^31^ or (2) glaucophane = jadeite + enstatite + quartz + H_2_O^32^. However, the chemical analyses of our sample after the electrical conductivity measurements at 1.5 GPa indicate enstatite, olivine, plagioclase, and melt (Fig. 3a). This disparity can be explained by the dissolution of quartz in the melt and olivine crystallization in consequence via reaction, enstatite = olivine + SiO_2_ (in melt)^33^. The plagioclase found in our sample appears to crystallize from the melt during the cooling of the sample. To preserve the mineral assemblages after the dehydration, in our experiment at 6 GPa, we limit the maximum temperature to 950 K. The breakdown of starting glaucophane sample results in jadeite, enstatite, coesite, and secondary amphiboles (cummingtonite and barroisite) (Supplementary Table S2). Omphacite has been observed surrounding jadeite grains (Fig. 3b).

## Discussion

The electrical conductivity of glaucophane before the dehydration is slightly lower than the electrical conductivity of naturally occurring Cl- and/or F-bearing amphiboles, and broadly comparable to the Na-rich plagioclase (albite)^34–36^. This signifies that crystal structure-bound Na^+^ may not be as mobile as the defect-bound volatiles or halogens. Na occupies both the alkali (A) and (B) sites in the crystal structure of amphiboles. Previous studies have shown that the preferential incorporation of Na in the large A sites may enhance the electrical conduction in amphiboles^37^. It appears that Na in glaucophane preferentially occupies the B-sites (0.02 mols in the A-site, 1.91 mols in the B-site), thus exerting negligible influence on the conductivity before dehydration.

**Figure 4:**
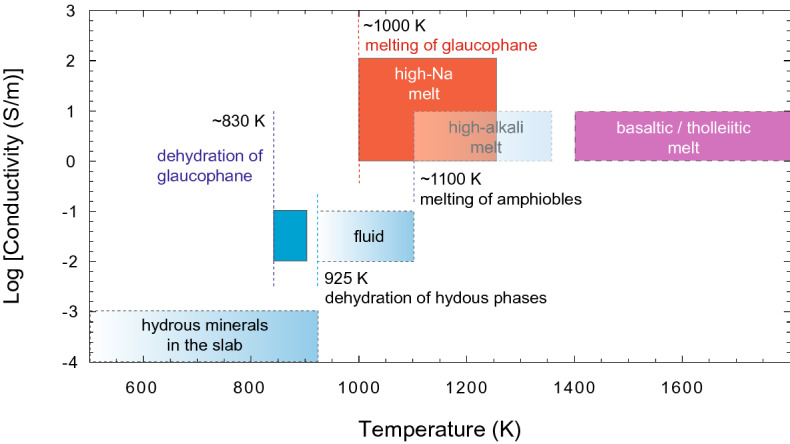
A compilation of electrical properties of hydrous minerals, fluid, and melt in the slab. The comparison of the electrical properties of hydrous minerals and their dehydration product (Manthilake et al. 2021a, 2021b^7,8^ and references therein) with glaucophane and its dehydration products. The dehydration and melting temperatures of glaucophane (gln) are lower than those observed for other major hydrous minerals in subduction zones.

The fluid released during the dehydration of glaucophane shows comparable electrical conductivity to that of aqueous fluids observed under subduction zone pressure and temperature conditions^26,38–41^ (Fig. 4). While Na^+^ can be mobile in fluids, the compatible nature of Na to glaucophane may restrict the partition of Na to the fluid phase during the dehydration^42^, thus limiting the role of Na^+^ as a charge carrier in the fluid phase. Compared to the electrical conductivity of the fluid at 1.5 GPa, the increase of electrical conductivity associated with dehydration of glaucophane at 6 GPa appears to be larger (Fig. 1). We attribute this observation to a higher fluid fraction in the sample or the higher ion concentrations, such as ionization of H_2_O in the supercritical fluid at the elevated pressure^26^.

**Figure 5:**
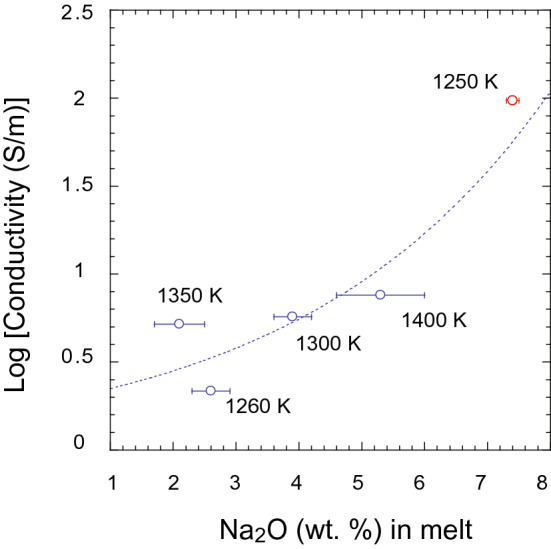
Electrical conductivity of silicate melt as a function of Na contents. The electrical conductivity of melts produced by dehydration and melting of amphiboles such as, ferri-kaersutite^7^, actinolite^7^, hastingsite^7^, tremolite^7^, and glaucophane (present study) at 1.5 GPa are plotted against the Na_2_O contents in the melt. The uncertainties in composition and electrical conductivities are smaller than the symbol size. The highest temperatures of electrical measurements are shown next to individual symbols.

Na appears to have a profound effect on the electrical conductivity of silicate melts. The melting of glaucophane increases the conductivity by a factor of ~ 100 compared to the conductivity of the aqueous fluid at 1.5 GPa (Fig. 1a). Our observation suggests that the electrical conduction in silicate melt, produced after dehydration of amphibole, is largely controlled by the mobility of Na^+^ ions (Fig. 5). This observation agrees well with previous experimental results, which identified Na^+^ as the principal charge carrier in basaltic and albite melts^30^. Compared to Na^+^, the effect of other major cations, such as Ca^+2^, Al^+3^, and Fe^+2/+3^, on the electrical conductivity is found to be negligible^7^. It has also been shown that the presence of H_2_O promotes the mobility of Na^+^ in silicate melts^30^. The Na-rich hydrous melt produced by the melting of glaucophane exhibits an electrical conductivity of 97 S/m at 1250 K. Extrapolation to high temperatures shows that the Na-rich melts occurring in the subduction zone settings are significantly more conductive than the silicate melts observed in other geological settings^43–45^ (Fig. 4). In contrast, the hydrous silicate melts produced by melting of tremolite, actinolite, ferri-kaersutite, and hornblende amphibole compositions show a convergence of electrical conductivities around 1 S/m above 1250 K (Fig. 5). These values closely resemble the electrical conductivity values obtained for hydrous basalts that are commonly found in volcanic-arc settings^43,46^ (Fig. 4).

### Geological significance of dehydration melting of glaucophane in the slab

The phase relations at the solidus of the subducted oceanic crust have been investigated by several studies^1,20,47^. Typically, amphiboles in the slab are thought to break down at pressures greater than 2.5 GPa. The dehydration reaction of amphibole starts at around 50 km depth in hot subduction systems and continues up to 75 km depending on the slab surface temperature at 2.5 GPa. The stability of amphibole could be extended to a depth beyond 75 km by substituting OH with F in the amphibole crystal structure^28,29^. In our 6 GPa experiment, glaucophane goes through a dehydration reaction forming jadeite + enstatite + quartz. We also observe the formation of secondary F-bearing amphibole (Supplementary Table S2) attesting to the stability of the amphibole phase at this pressure condition. The crystallization of F-bearing amphiboles has also been demonstrated by other amphibole dehydration experiments conducted under subduction zone pressure and temperature conditions^7^.

### Electrical conductivity of glaucophane as a geothermometer

**Figure 6:**
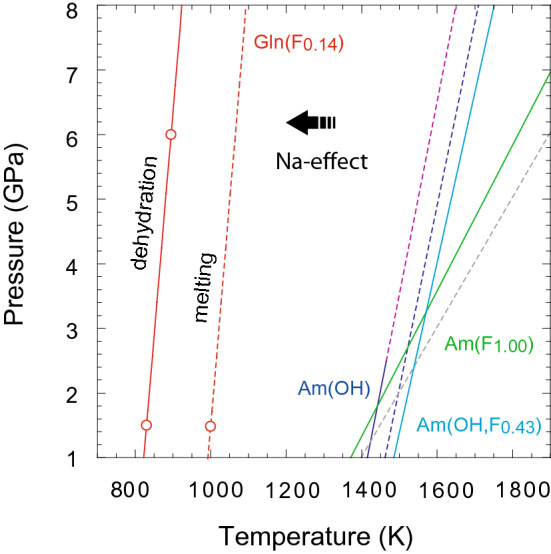
The stability of glaucophane at high-pressure and high-temperature. The solid red line indicates a linear fit through experimental data points obtained at 1.5 and 6 GPa. The red dashed line is drawn parallel to the dehydration curve through the melting temperature obtained at 1.5 GPa. The dark blue, light blue, and green lines are the dehydration and melting temperatures observed for F and Na-free amphibole^29^, Na-free amphibole with 0.43 mol F replacing (OH^−^)^28^, and Na-free amphibole with 1 mol F^29^ at the pressure range from 3.0 to 5.5 GPa, respectively. *am *amphibole; *gln* glaucophane.

**Figure 7:**
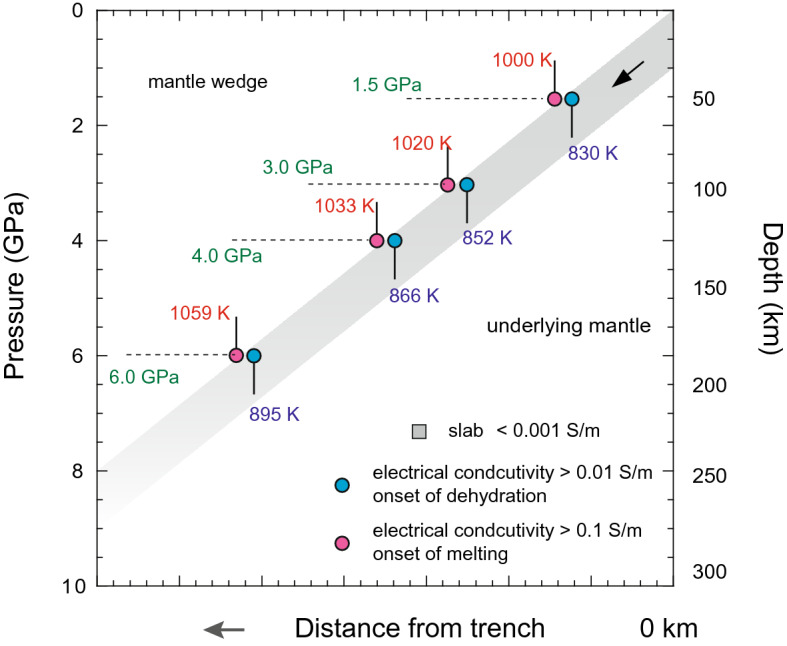
The electrical conductivity based geothermometer. The locus of dehydration of glaucophane in the slab is shown in blue circles and the locus of melting of glaucophane is shown in pink circles. The characteristic electrical conductivity at dehydration of glaucophane is 0.01 S/m, the electrical conductivity at the onset of melting of glaucophane is 0.1 S/m. In comparison, the electrical conductivity of the hydrous slab is about 0.001 S/m. The corresponding slab surface temperatures are shown at different pressures.

The dehydration temperature we observed for glaucophane using the in situ electrical conductivity measurements appears to occur at lower temperatures compared to the decomposition of OH^−^ and F^−^ bearing amphiboles^28,29^ (Fig. 6). We attribute this decrease in dehydration and melting temperature to the presence of Na. Albite, another principal host of Na in the slab, transforms to jadeite + quartz through a fluid absent reaction at high pressure and temperature^48,49^, thus, may not influence the electrical conductivity measurements. The low dehydration and melting temperature associated with the high electrical conductivity makes glaucophane an ideal indicator-mineral for tracking the slab surface temperature in subduction zones (Fig. 7).

As a case study, we investigated the 2D magnetotelluric (MT) profile at the Sodo ridge, where the old and cold Pacific plate subducts beneath North-East Japan^50,51^. The MT profile of the Sodo ridge provides a case for the extreme scenario found in subduction systems, where the high electrical anomaly appears to occur at depths greater than 150 km. The conductivity-depth profile at the Sodo ridge displays an increase of electrical conductivity from 0.01 to 0.1 S/m at the slab surface at 150–170 km depths, which is the characteristic electrical conductivity for aqueous fluids^8,26^. This apparent dehydration event is followed by a further increase of conductivity to more than 1 S/m at the slab surface at a depth of 185 km^50^. Given that glaucophane breaks down at relatively low temperatures, and any other types of melting in the slab cannot occur before the melting of glaucophane, we consider that the extremely high conductivity displayed at 185 km depth may be related to the melting of F-bearing glaucophane and the subsequent release of Na-rich melt in the slab.

**Figure 8:**
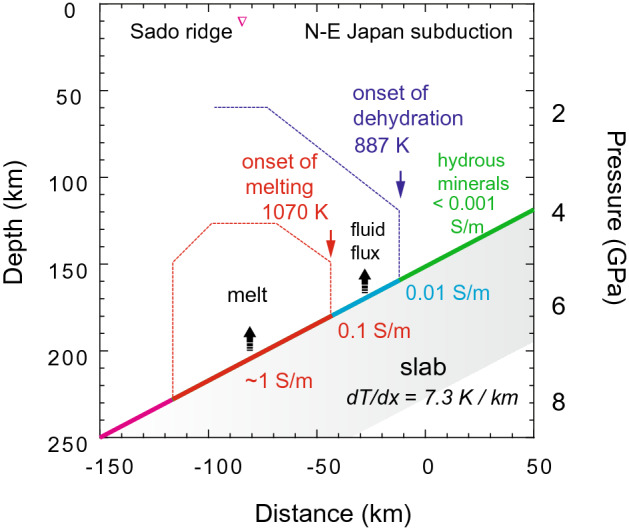
Application of the glaucophane geothermometer to N–E Japan subduction zone. Two temperature-fixed points for the N–E Japan subduction slab are determined using the electrical conductivity model discussed in Fig. 6. The electrical conductivity structure of the subduction system is taken from Toh et al. 2006^50^. The green, blue, and red zones were determined based on the electrical conductivity of the wedge mantle adjacent to the slab surface. The areas marked by dashedlines indicate high conductivity regions in the mantle wedge.

Assuming that incorporation of F stabilizes amphiboles below depths of 75 km and the high conductivity anomaly is caused by the melting of F- bearing glaucophane; we can estimate the depth where the blueschist-eclogite transition happens in the slab. In the case of Sodo ridge, the blueschist-eclogite transition can be fixed at a depth of 160 km (~ 5.5 GPa) and a temperature of ~ 887 K (Fig. 8). By setting the solidus of glaucophane parallel to the dehydration curve, we could estimate the onset of melting at 185 km depth (~ 6.5 GPa) and 1070 K. Our calculations suggest a thermal gradient of 7.3 K/km along the slab surface for the North-East Japan subduction zone. We conclude that the precise temperature for slab surfaces can be derived from the phase relations of glaucophane. These estimates can benchmark the slab-surface temperatures and provide a temperature-fixed point for thermal models of subduction zones.

## Methods

Finely ground powders of glaucophane (from Sesia–Lanzo Zone, Aosta Valley, Western Alps) were prepared from inclusion-free natural crystals, handpicked under a binocular microscope. The chemical composition of glaucophane was determined before the electrical conductivity measurements by electron probe microanalysis using a Cameca SxFiveTactis electron microprobe operating at an accelerating voltage of 15 kV and a beam current of 20 nA. The composition of the starting glaucophane is reported in Supplementary Table 1.

High pressure–temperature experiments were performed using a 1500-ton multi-anvil apparatus installed at the Laboratoire Magmas et Volcans. The experiments were performed in an 18/11 assembly, with an 18 mm edge length Cr_2_O_3_-doped MgO octahedral pressure medium and 11 mm WC anvil truncation edge length (Supplementary Figure S1). Cylindrical sample specimens for electrical conductivity measurements were synthesized by hot pressing of amphibole power in rhenium (Re) capsule at 1.5 GPa and 700 K for 1 h. Pre-sintered cylindrical samples were placed in a polycrystalline MgO capsule, which electrically insulated the sample from the furnace during the measurements. Two Ni disks placed at the top and bottom of the sample served as electrodes for the electrical conductivity measurements. These Ni disks also served as an oxygen buffer, controlling the oxygen fugacity in the sample close to the Ni-NiO buffer. A W_95_Re_5_–W_74_Re_26_ thermocouple junction was placed at one side of the sample, which monitored the temperature. One cable formed the thermocouple, and a separate W_95_Re_5_ cable was placed at the opposite side of the sample, connected to the impedance spectroscopy for the electrical conductivity measurements. MgO ceramic sleeves insulated the electrode wires from the furnace. All ceramic assembly parts, including the pressure media, were baked at 1273 K for more than 12 h and stored at 400 K in high-vacuum furnaces (< 100 mTorr) before assembling. This step reduced the exposure of assembly components to atmospheric moisture and other impurities.

The electrical conductivity of glaucophane samples was determined at pressures of 1.5 and 6 GPa, based on the impedance spectroscopy method with a Modulab MTS Impedance gain-phase analyzer in the frequency range of 10^6^–10^1^ Hz. Sample resistance was measured in 30–50 K temperature steps. The maximum temperatures attained were 1258 K at 1.5 GPa and 950 K at 6 GPa.

Polycrystalline samples can be characterized by a combination of resistor–capacitor (R–C) or resistor-constant phase element (R-CPE) circuits, and the resistance can be obtained by fitting the impedance spectra to appropriate equivalent circuits. Once the sample resistance has been determined, conductivity can be calculated using the sample diameter and length measured after each experiment, assuming the sample geometry remained unchanged during the experiment. The activation enthalpy ($\Delta H$) of each conduction mechanism can be obtained by fitting the data to the Arrhenius equation, $\sigma ={\sigma }_{0}exp\left(-\Delta H|RT\right)$, where σ is the electrical conductivity (S/m), $T$ is the absolute temperature, ${\sigma }_{0}$ is the pre-exponential factor (S/m), and $R$ is the gas constant (J/K/mol).

After an electrical conductivity experiment, cross-sections of the experimental run products were investigated using an electron probe micro analyzer and energy dispersive X-ray spectroscopy (EDS) chemical mapping with a JEOL JSM-5910LV scanning electron microscope, to identify mineral assemblages and the dehydration reaction products. The compositions of the fluid and melt phases were determined by mass-balance calculations based on the mineral proportions and their chemical compositions of recovered samples after the dehydration/melting events^7^.

## Supplementary Information

Supplementary Information.

## Data Availability

All data generated or analyzed during this study are included in this article. The raw electrical conductivity data are available from the corresponding author on reasonable request.
