# Supplementary material for: Tracking slab surface temperatures with electrical conductivity of glaucophane
Source: Sci Rep. 2021 Sep 9;11:18014. doi: 10.1038/s41598-021-97317-0 (PMC8429578; doi:10.1038/s41598-021-97317-0)
Supplement: Supplementary file 1 — Supplementary Information. [file 41598_2021_97317_MOESM1_ESM.pdf]

Supplementary Information

Tracking slab surface temperatures with electrical conductivity of glaucophane

Geeth Manthilake[1*], Ye Peng[2], Kenneth T. Koga[1], Mainak Mookherjee[2]

[1] Laboratoire Magmas et Volcans CNRS, IRD, OPGC, Université Clermont Auvergne, 63000 Clermont-Ferrand, France

[2] Earth Materials Laboratory, Department of Earth, Ocean and Atmospheric Sciences, Florida State University, Tallahassee, FL, 32306, USA

*Corresponding author: geeth.manthilake@uca.fr

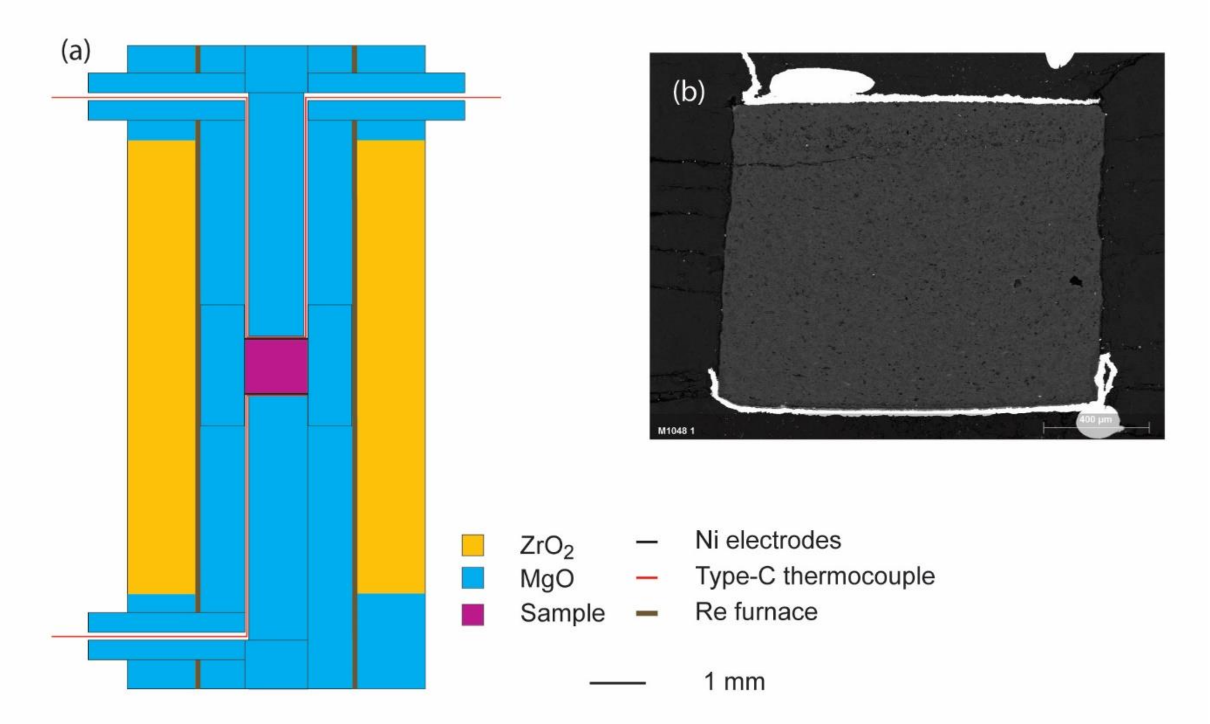

Supplementary Figure S1. (a) Schematic diagram showing the cross section of the high-pressure cell assembly for electrical conductivity measurements. (b) A backscattered electron (BSE) image showing the cross section of the sample after electrical conductivity measurements.

Supplementary Table 1. Composition of mineral phases after dehydration and melting at 1.5 GPa

Phase	Glaucophane*	Enstatite	Plagioclase	Olivine	Melt	Melt#
**SiO_2**	55.9(9)	56.3(10)	60.0(7)	40.2(5)	59.5(6)	57.3(3)
**TiO_2**	0.03(5)	0.2(2)	0.12(6)	0.04(4)	0.07(0)	0.02(3)
**Al_2O_3**	4.7(20)	2.9(16)	24.1(7)	0.12(5)	21.1(2)	3.2(1)
**FeO**	20.2(30)	7.3(1)	0.7(6)	13.1(7)	1.08(2)	22.98(8)
**MnO**	0.1(1)	0.38(4)	0.03(3)	0.28(3)	0.16(2)	0.08(6)
**MgO**	8.5(9)	27.6(45)	0.5(6)	45.2(3)	0.1(0)	6.6(2)
**CaO**	0.55(18)	4.4(4)	6.4(5)	0.19(1)	1.84(4)	0.00
**Na_2O**	6.8(2)	0.4(4)	7.9(3)	0.02(1)	3.2(2)	7.38(8)
**K_2O**	0.03(1)	0.00	0.03(4)	0.00	0.1(0)	0.04(6)
**F**	0.0030(2)	0.00	0.00	0.00	0.004(10)	0.00
**Cl**	0.005(1)	0.00	0.00	0.00	0.006(10)	0.00
**H_2O^+**	2.1(6)	0.00	0.00	0.00	3.9(6)	2.5(1)
**Total**	**98.92**	**99.48**	**99.78**	**99.15**	**91.06**	**100.1**

Measurements by an electron microprobe are reported here. Values in parentheses represent one standard deviation on the averages in terms of the smallest unit cited. For example, 55.9(9) should read as 55.9 +/- 0.9. * Starting glaucophane. # Melt compositions are determined by a modified mass-balance procedure (Wu and Koga 2013). H_2O^+ values are determined by the stoichiometric balance of amphibole composition following the procedure of Locock (2014).

Supplementary Table 2. Composition of mineral phases after dehydration of glaucophane at 6.0 GPa

Phase	Glaucophane*	Jadeite	Enstatite	Omphacite	Coesite	Secondary Amphibole
**SiO_2**	55.9(9)	60.0(1)	56.3(10)	58.3(7)	97.6(18)	44.4(36)
**TiO_2**	0.03(5)	0.2(1)	0.2(2)	0.26(1)	0.05(2)	0.14(4)
**Al_2O_3**	4.7(20)	19.5(2)	2.9(16)	12.3(15)	1.4(4)	26.3(27)
**FeO**	20.2(30)	2.41(5)	7.3(1)	5.5(16)	0.4(1)	6.8(10)
**MnO**	0.1(1)	0.1(0)	0.38(4)	0.24(2)	0.01(1)	0.31(6)
**MgO**	8.5(9)	2.9(2)	27.6(45)	12.2(26)	1.3(8)	13.9(19)
**CaO**	0.55(18)	4.1(4)	4.4(4)	5.1(51)	0.27(4)	2.2(14)
**Na_2O**	6.8(2)	12.3(2)	0.4(4)	6.9(7)	0.4(2)	1.5(19)
**K_2O**	0.03(1)	0.0(0)	0.00	0.01(0)	0.01(1)	0.00(0)
**F**	0.0030(2)	0.00	0.00	0.00	0.00	0.05(3)
**Cl**	0.005(1)	0.00	0.00	0.00	0.00	0.01(1)
**H_2O^+**	2.1(6)	nd	nd	nd	nd	nd
**Total**	**98.92**	**101.5**	**99.48**	**100.93**	**101.5**	**95.5**

Measurements by an electron microprobe are reported here. Values in parentheses represent one standard deviation on the averages in terms of the smallest unit cited. For example, 55.9(9) should read as 55.9 +/- 0.9. * Starting glaucophane. nd represents "not determined".
